# Acute mucosal pathogenesis of feline immunodeficiency virus is independent of viral dose in vaginally infected cats

**DOI:** 10.1186/1742-4690-7-2

**Published:** 2010-01-19

**Authors:** Kristina E Howard, Stacie K Reckling, Erin A Egan, Gregg A Dean

**Affiliations:** 1 Center for Comparative Medicine and Translational Research, Department of Molecular Biomedical Sciences, College of Veterinary Medicine, North Carolina State University, Raleigh, NC, 27606, USA; 2 Current address: Immunobio, 920 Main Campus Drive, Suite 405, Raleigh, NC, 27606, USA

## Abstract

**Background:**

The mucosal pathogenesis of HIV has been shown to be an important feature of infection and disease progression. HIV-1 infection causes depletion of intestinal lamina propria CD4+ T cells (LPL), therefore, intestinal CD4+ T cell preservation may be a useful correlate of protection in evaluating vaccine candidates. Vaccine studies employing the cat/FIV and macaque/SIV models frequently use high doses of parenterally administered challenge virus to ensure high plasma viremia in control animals. However, it is unclear if loss of mucosal T cells would occur regardless of initial viral inoculum dose. The objective of this study was to determine the acute effect of viral dose on mucosal leukocytes and associated innate and adaptive immune responses.

**Results:**

Cats were vaginally inoculated with a high, middle or low dose of cell-associated and cell-free FIV. PBMC, serum and plasma were assessed every two weeks with tissues assessed eight weeks following infection. We found that irrespective of mucosally administered viral dose, FIV infection was induced in all cats. However, viremia was present in only half of the cats, and viral dose was unrelated to the development of viremia. Importantly, regardless of viral dose, all cats experienced significant losses of intestinal CD4+ LPL and CD8+ intraepithelial lymphocytes (IEL). Innate immune responses by CD56+CD3- NK cells correlated with aviremia and apparent occult infection but did not protect mucosal T cells. CD4+ and CD8+ T cells in viremic cats were more likely to produce cytokines in response to Gag stimulation, whereas aviremic cats T cells tended to produce cytokines in response to Env stimulation. However, while cell-mediated immune responses in aviremic cats may have helped reduce viral replication, they could not be correlated to the levels of viremia. Robust production of anti-FIV antibodies was positively correlated with the magnitude of viremia.

**Conclusions:**

Our results indicate that mucosal immune pathogenesis could be used as a rapid indicator of vaccine success or failure when combined with a physiologically relevant low dose mucosal challenge. We also show that innate immune responses may play an important role in controlling viral replication following acute mucosal infection, which has not been previously identified.

## Background

The recent failure of the STEP clinical trial of the MRKAd5 HIV-1 gag/pol/nef vaccine has raised important questions about vaccine development for HIV-1[1-3]. Participants in the Phase I trial had robust measurable T cell responses to vaccination [4]; similar robust T-cell responses were observed in participants in the Phase IIB trial, however, they afforded no protection against HIV-1 infection as compared to the control group [5]. These data suggest that measurable *in vitro *T cell responses of the participants were not a reliable predictor of vaccine protection. Identification of appropriate and reliable correlates of protection has been elusive in pathogenesis and vaccine studies. Many potential immunologic correlates have been suggested including cytotoxic CD8+ T cells, neutralizing antibodies, and preservation of memory and effector lymphocyte populations in the gastrointestinal mucosa [6]. However, numerous studies examining the role of T cell and antibody responses in the protection of highly-exposed persistently seronegative (HEPS) individuals, and control of viral replication in elite controllers (EC) and long-term non-progressors (LTNP) [7-12], have yielded conflicting results [13-16].

Collectively, these observations raise new questions about defining correlates of protection and how they could be more clearly distinguished in the context of future vaccine trials [17,18]. Further, as animal model vaccine trials appeared to show the MRKAd5 vaccine to be protective [19-23], the design and assumptions used in animal model vaccine trials might also need to be reconsidered.

Vaccine studies using animal models often employ high doses of challenge virus to ensure a high viral set point in control animals so that a reduction of viral burden in vaccinated animals can be used as an indicator of efficacy. Unfortunately, high challenge doses do not mimic natural infection and could lead to flawed conclusions about the true efficacy of a vaccine [24]. The majority of HIV-1 infections occur via the mucosal route [25]. Certain studies suggest that infection can occur in serodiscordant couples with repeated sexual exposure from their HIV-1 positive partners who have plasma viral loads ranging from 5-400 copies/ml [26]. These observations suggest that high viral challenge doses are not physiologically relevant in natural HIV transmission. Further, animal model studies have shown that low dose infection can result in either productive or latent infection [27-31]. In contrast, several investigators have suggested that low viral dose may be partially responsible for individuals who are either HEPS or LTNP [32-34]. Importantly, the effect of initial viral dose on the presence and severity of mucosal pathogenesis is unclear, in particular when the route of infection is via the reproductive mucosa. Therefore, a better understanding of the relationship between viral dose, mucosal pathogenesis and mucosal immune response would enhance our ability to design and interpret vaccine trials.

In the present study, we employed the well-described cat/feline immunodeficiency virus (FIV) model [35-39] to investigate the relationship between viral dose and immune pathogenesis. We vaginally challenged three groups of cats with different infectious doses of cell-associated and cell-free FIV to determine the effect of viral dose on mucosal leukocyte populations. To address possible correlates of protection, we assessed the role of innate, cell-mediated, and humoral immune responses in acute FIV infection to determine if any of these immune responses were associated with decreased viral dissemination and protection of the gastrointestinal mucosa. These studies provide new insight into early mechanisms of control over viral replication, with particular emphasis on the responses in the mucosa.

## Results

### Viral load

**Figure 1:**
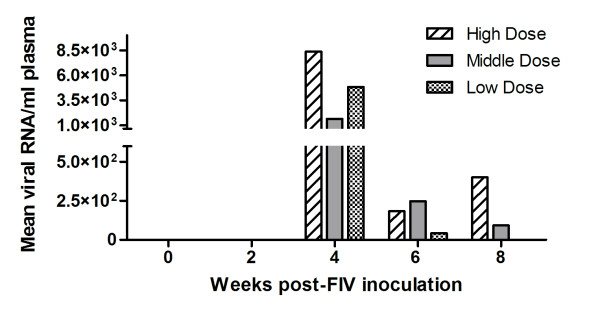
**Mean plasma viral RNA**. Plasma from blood sampled at weeks 0, 2, 4, 6 and 8 post-infection was evaluated for viral RNA using real-time PCR. Mean viral RNA copies per ml of plasma are shown for high, middle and low dose groups.

Cats vaginally infected with high, middle, or low doses of cell-associated and cell-free virus were evaluated for viral load by PCR and virus isolation. Figure 1 summarizes mean plasma viral load for each group. Peak viremia was detected at four weeks post-infection in all groups, with plasma viremia detected in 4/6 high (range, 2.2 × 10^4 ^to 2.0 × 10^3 ^copies/ml plasma), 3/6 middle (range, 1.8 × 10^4 ^to 1.5 × 10^2 ^copies/ml plasma) and 2/5 low (range, 2.4 × 10^4 ^to 1.7 × 10^2 ^copies/ml plasma) dose inoculated cats. Provirus in PBMC was detected in 3/6 high, 1/6 middle and 2/5 low dose inoculated cats. Virus was isolated from unfractionated bone marrow in 5/6 high, 5/6 middle, and 5/5 low dose inoculated cats. By at least one of these measures, each cat, regardless of inoculum dose, was infected with FIV. Interestingly, although shown to be infected, eight cats did not have detectible plasma viremia.

### Effect of FIV infection on CD4+ and CD8+ T cell numbers

**Figure 2:**
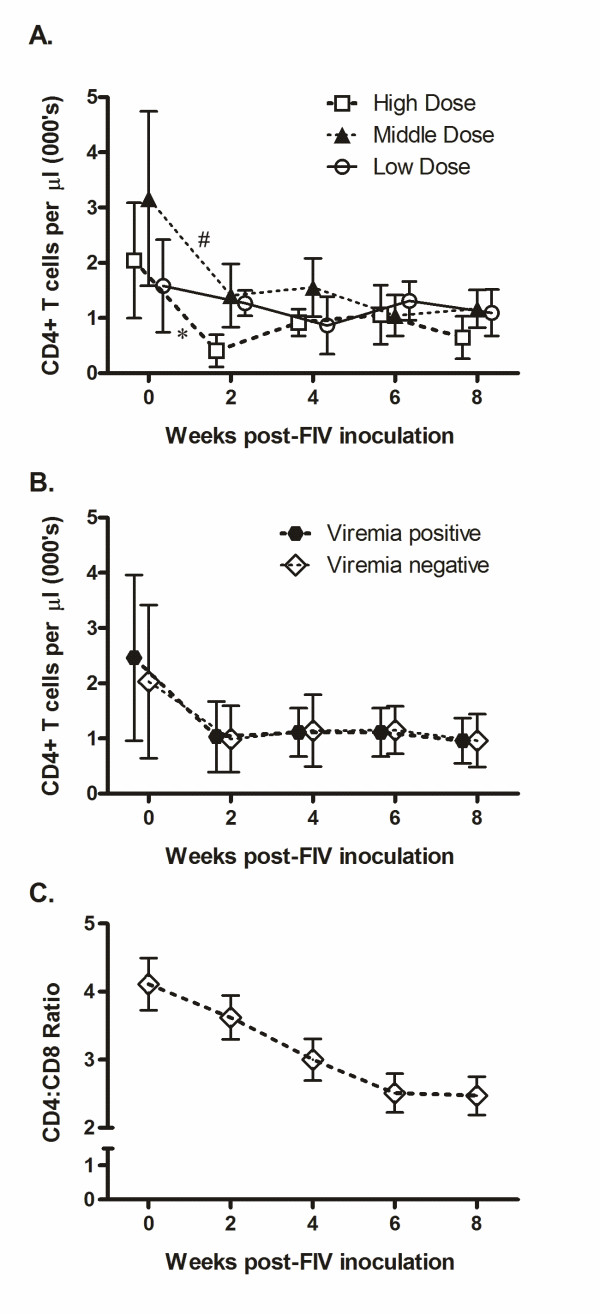
**Absolute CD4+ T cell count in PBMC**. Blood sampled at weeks 0, 2, 4, 6 and 8 post-infection was evaluated for phenotypic expression of CD4+ T cells and calculated based on total WBC counts with differential cell count assessed from cytological evaluation. Mean and standard deviation for absolute CD4+ T cell counts are presented for high, middle and low dose groups (A), and based on presence or absence of viremia (B). Mean and standard errors for the CD4:CD8 ratio are shown for all dose groups combined (C). Statistics were calculated using ANOVA comparing baseline week with post-infection samples within each group. Significance shown using p-values, with * p < 0.01 and # p = 0.06.

Two weeks post-infection, absolute CD4+ T cell numbers in PBMC (Figure 2A) were decreased in the high (p = 0.002) and middle (p = 0.06) dose groups, whereas the low dose group experienced a modest decrease by four weeks post-infection that was not significant. All three groups had comparable mean CD4+ T cell numbers at study end; mean levels which were lower than pre-infection levels. Importantly, the presence or absence of viremia was not correlated with absolute CD4+ T cell numbers (Figure 2B). All dose groups had similar reductions in CD4:CD8 ratio over the course of the study (Figure 2C).

**Figure 3:**
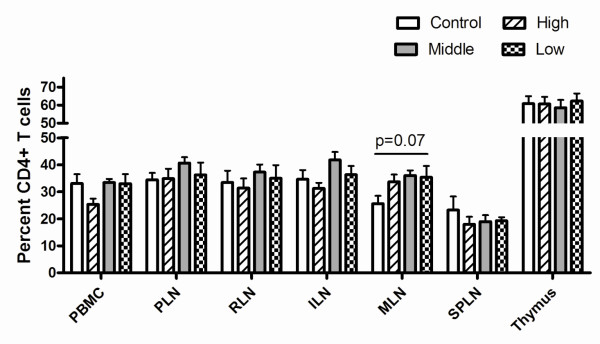
**Percent CD4+ T cells in peripheral sites eight weeks post-infection**. The percent CD4+ T cells is based upon total mononuclear cells isolated. Mean and standard deviation are shown for control, high, middle and low dose groups at euthanasia. No statistically significant differences were identified using ANOVA analysis. PBMC, peripheral blood mononuclear cells; PLN, prescapular lymph node; RLN, retropharyngeal lymph node; ILN, medial iliac lymph node; MLN, mesenteric lymph node; and SPLN, spleen.

**Figure 4:**
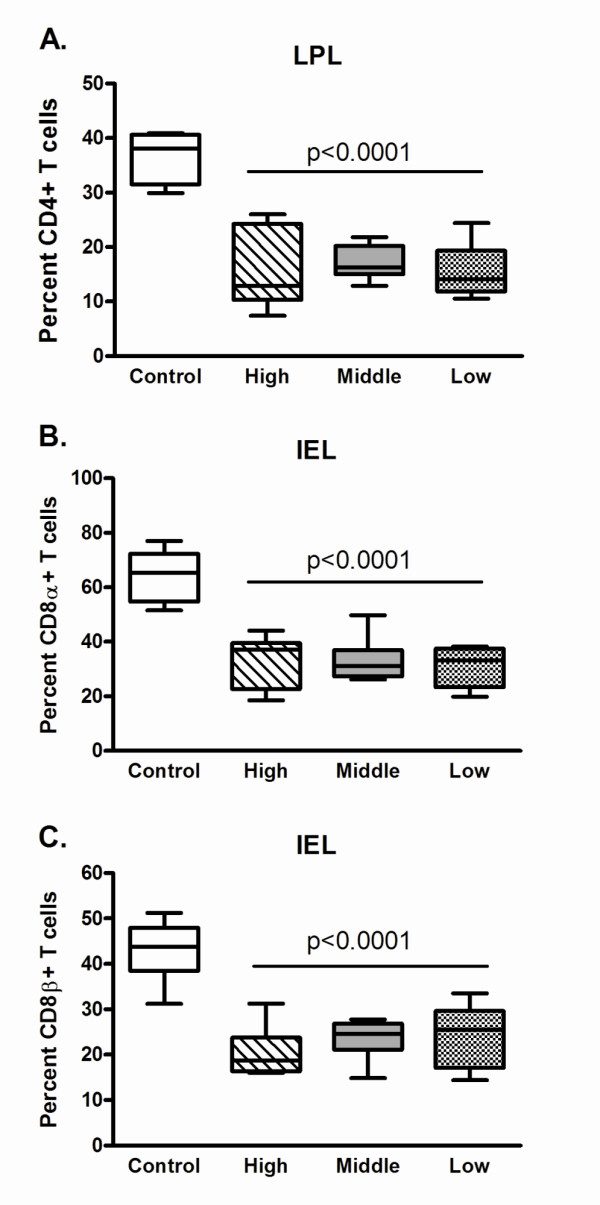
**Alterations in mucosal lymphocyte populations eight weeks post-infection**. Box and whisker plots show the median with upper and lower quartile represented by the boxes, and minimum and maximum values shown by the whiskers. Percent of CD4+ T cells in LPL (A), CD8α+ T cells in IEL (B), and CD8β+ T cells in IEL (C) are shown for control, high, middle and low dose groups at euthanasia. Statistical significance was calculated using ANOVA, with p-value shown for FIV-infected groups compared to controls.

No significant changes in CD4+ T cell percentages were observed in PBMC, lymph nodes, spleen or thymus for any group (Figure 3), however, a significant decrease in LPL CD4+ T cell percentages, averaging a 57% loss as compared to control cats, was noted regardless of inoculum dose (Figure 4A). The loss of CD4+ LPL was further magnified considering a significant loss in total yield of LPL occurred (mean control LPL yield was 6.80 × 10^7 ^vs. FIV-infected LPL yield of 3.94 × 10^7^, p = 0.00037). Furthermore, a significant decrease in the percentage of CD8α+ and CD8β+ T cells was found in IEL from all dose groups as compared to controls (Figure 4B,C). As CD8αα+ and CD8αβ+ T cells serve different functions in the epithelial compartment, we also assessed if either of these populations was specifically lost. CD8αβ+ T cells were significantly decreased in all dose groups, compared to controls (ANOVA, p < 0.001). CD8αα+ T cell percentages were not significantly decreased in FIV-infected cats (p = 0.1311), however, the overall trend showed decreased percentages in FIV-infected cats [18.11%] versus controls [24.85%].

### Innate immune response

**Figure 5:**
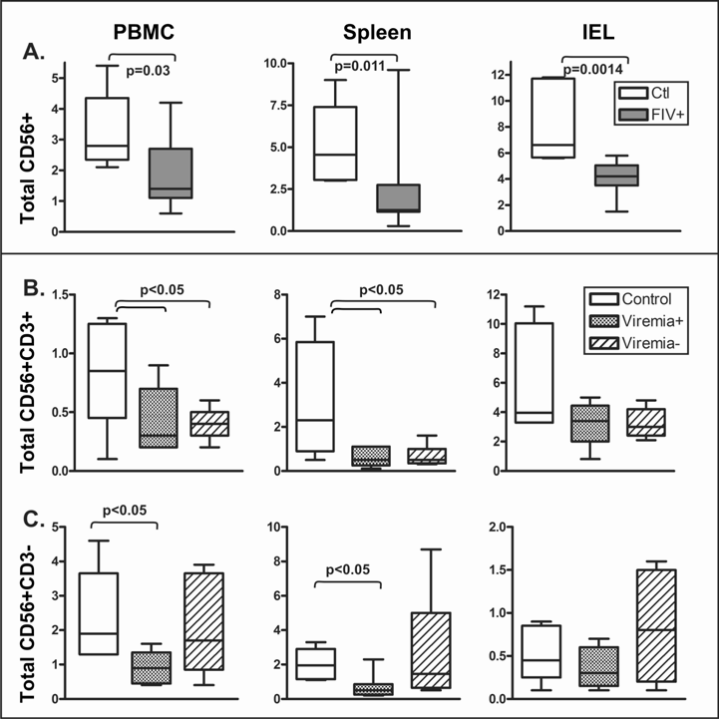
**NK cell subsets eight weeks post-infection**. Percent NK cells (CD56+) in PBMC, Spleen and IEL for all dose groups of FIV-infected cats versus control cats (A). Percent of NKT cells (B) and classic NK cells (C) are shown based presence or absence of viremia versus control cats. Box and whisker plots show the median with upper and lower quartile represented by the boxes, and minimum and maximum values shown by the whiskers. Statistics were calculated using an unpaired t-test in (A), and based on ANOVA in (B) and (C), with significance shown using p-values where differences were identified.

While all study cats were infected with FIV, a few cats in each dose group did not become viremic. To identify immunologic populations that might mediate the apparent control of viremia, we assessed total NK cells (CD56+CD3+/-), classic NK cells (CD56+CD3-) and NKT cells (CD56+CD3+) in blood, draining lymph node (data not shown), spleen, and IEL. Total CD56+ NK cell expression was significantly decreased in FIV-infected cats as compared to control cats in each site at eight weeks post infection (Figure. 5A). Figure 5(B) and 5(C) show NKT cell and classic NK cell percentages, respectively, in viremic, non-viremic and control cats. NKT cell percentages were significantly reduced in PBMC and spleen from FIV-infected cats, regardless of viremia status. In contrast, CD56+CD3- NK cells were significantly decreased only in viremic cats.

### Cell-mediated immune responses

**Figure 6:**
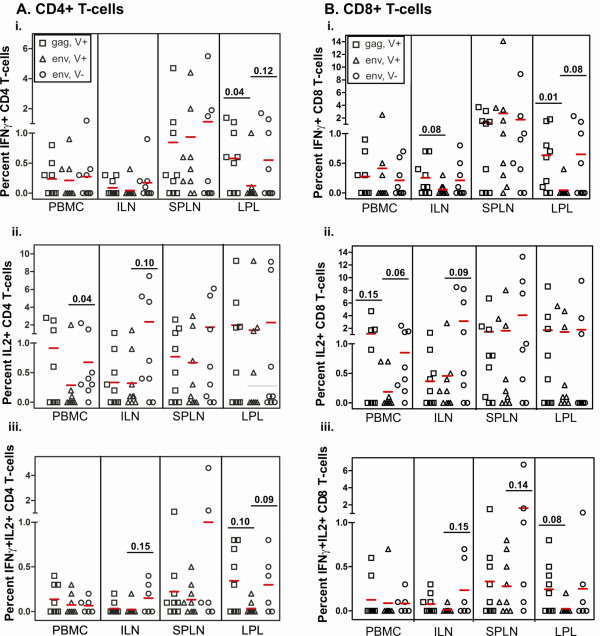
**Intracellular cytokine expression of IFNγ and IL-2 by CD4+ and CD8+ T cells eight weeks post-infection**. Expression of IFNγ is shown in the top panels, IL-2 in the middle panels and dual expressing (IFNγ+IL-2+) in the bottom panels for CD4+ T cells (A) and CD8+ T cells (B). Individual data points are shown with mean expression indicated by a line for each data set. Significant differences in cytokine expression are indicated by a line over the applicable data sets and p-value indicated. Calculations were made using an unpaired t-test.

Anti-Gag and anti-Env specific CD4+ and CD8+ T cell responses were assessed in PBMC, peripheral and draining lymph nodes, spleen, IEL and LPL (Figure. 6 and data not shown). Cells were stimulated for six hours with peptide pools for Gag and Env [40], and then intracellular IL-2, IFNγ, and TNFα production was determined in CD4+ and CD8+ T cells. No differences in cytokine production were found when comparing groups based on inoculum dose (data not shown). Differences in IFNγ and IL-2 production were compared on the basis of presence or absence of viremia.

Responses in viremic cats tended to be directed to Gag rather than Env peptides, whereas non-viremic cats had a similar magnitude of response to both Gag (data not shown) and Env peptides. Significant differences were noted in viremic cats when comparing the production of IFNγ by CD4+ and CD8+ T cells from the lamina propria in response to Gag versus Env peptides. This trend was also evident in CD4+ and CD8+ LPL, which produced both IL-2 and IFNγ against Gag peptides, but not Env peptides.

FIV-infected, non-viremic cats were more likely to produce cytokines in response to Env peptide stimulation as compared to viremic cats. This was evident in CD4+ and CD8+ IFNγ specific responses in LPL, IL-2 responses in the draining lymph node (ILN), and IL-2+IFNγ+ producing cells in the ILN. Compared to viremia positive cats, non-viremic cats also showed significant differences in anti-Env responses in CD4+ IL-2 producing PBMC. CD8+ IL-2 producing PBMC also showed a marked difference (p = 0.06). The trend of anti-Env responses was also identified in CD4+IL-2+IFNγ+ LPL and CD8+IL-2+IFNγ+ splenocytes.

### Humoral immune responses

To understand the contribution humoral immunity may have played in control of viremia, we assessed anti-Gag and anti-Env responses in serum and vaginal wash samples using a highly sensitive chemiluminescent ELISA assay. Two cats did not produce antibodies against either Gag or Env, three cats produced antibodies only to Env, and six cats produced anti-Gag antibodies at levels that would not be detectable using a commercial diagnostic test for FIV. The remaining six cats produced substantial titers to Gag and/or Env (Table 1). Almost all Gag and Env specific viral titers in vaginal wash were less than 1:256 or below the limit of detection for either IgA or IgG. Thus, using our highly sensitive ELISA, 88% of the cats seroconverted.

**Table 1:** Anti-p24 and anti-Env IgA and IgG antibody responses

		Vaginal IgA		Vaginal IgG		Serum IgG	
Group	Cat	Gag^1^	Env	Gag	Env	Gag	Env
High Dose	IQW3	128	128	0	32	1,048,576	262,144
	IQX7	128	0	0	0	4,096	512
	IRB5	0	0	64	0	0	512
	IRE5	0	0	0	256	262,144	2,097,152
	IRI4	64	64	64	0	4,096	0
	IRK4	64	32	32	0	2,048	128
							
Middle Dose	IQT2	0	0	0	0	0	0
	IQW5	0	0	64	64	131,072	524,288
	IQX6	0	0	256	0	1,024	0
	IRB4	0	32	0	0	1,024	512
	IRE3	0	0	0	0	0	512
	IRK5	1,024	32	256	0	131,072	64
							
Low Dose	IQT3	32	0	0	0	0	0
	IQW4	4,096	128	256	128	262,144	2,097,152
	IRB3	32	32	0	0	0	1,024
	IRK3	0	0	0	0	65,536	0
	IRK6	128	64	256	0	4,096	512

^1 ^Endpoint titer.

### Immune responses associated with control of viremia

**Figure 7:**
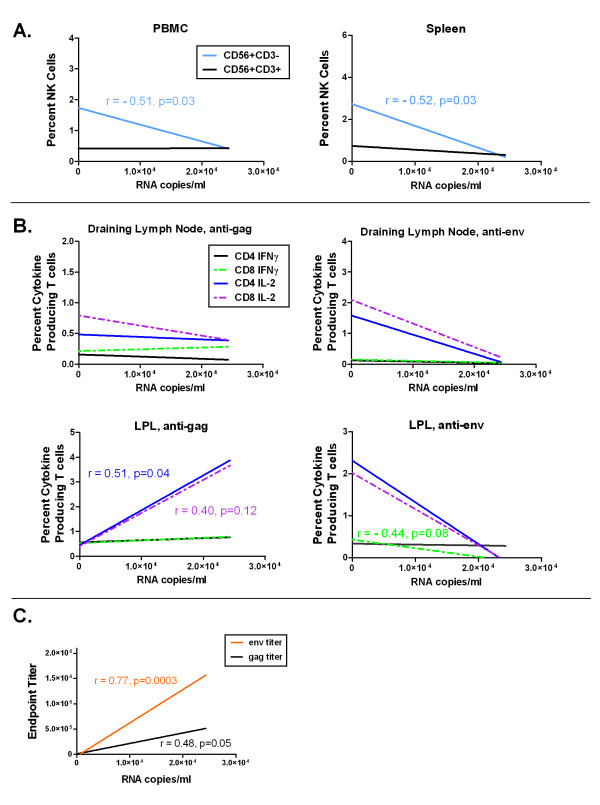
**Correlation of immune responses with viremia**. Innate, cell-mediated and humoral immune responses were correlated with peak viremia to determine if specific immune responses could be responsible for control of viremia. Shown are innate immune responses by CD56+CD3- and CD56+CD3+ NK cells in PBMC and spleen (A), cell-mediated responses by CD4+ and CD8+ T cells in draining lymph node and LPL (B), and humoral response using endpoint Env and Gag titers (C). Spearman correlations are shown, with p-values indicated.

Given the trends identified for innate, cell-mediated and humoral responses, we next determined if any of these responses correlated with control of viremia. In Figure 7, Spearman correlations are shown for NK cell subsets (A), draining lymph node and LPL production of IL-2 and IFNγ by CD4 and CD8 T cells (B), and serum Gag and Env titers (C).

Significant inverse correlations to viremia were identified in both PBMC and Spleen CD56+CD3- NK cells, with r = -0.51 and r = -0.52 respectively (Figure 7A). Cell mediated responses in the draining lymph node suggested a trend associated with anti-Env responses in CD4 and CD8 T cells producing IL-2, however this was not significant. Surprisingly, a significant positive correlation with viremia was identified for LPL CD4 T cells producing IL-2 in response to Gag (r = 0.51). Additional trends suggested T cell responses to Env in LPL may be associated with viral control; however, these correlations were not significant (Figure 7B). Antibody titers significantly correlated with the presence and magnitude of viremia, with anti-Env (r = 0.77) and anti-Gag (r = 0.48). These correlations indicate that higher serum titers to Env (and Gag to a lesser degree) positively correlated with the degree of peak viremia (Figure 7C).

## Discussion

Parameters of viral challenge are an important consideration in animal model pathogenesis and vaccine studies. The majority of human HIV-1 infections occur via the reproductive mucosa and frequently involve cell-associated and cell-free forms of virus [41-43]. Further, leukocyte numbers present in a single human semen sample can range from 1.0 × 10^4 ^to 1.0 × 10^8^, resulting in a potentially significant number of HIV-infected leukocytes in seminal fluid [44-46]. The biological relevance of cell-free, high viral dose inocula administered intravenously or intrarectally is questionable [24]. A goal of the present study was to mimick the inoculum diversity found during natural HIV infection and to administer the inoculum via the reproductive mucosa. Results showed that a relatively low dose of cell-free and cell-associated virus administered vaginally caused infection in all, yet viremia in only half of the cats. Moreover, viral dose was unrelated to the development of viremia. Importantly, regardless of the presence of viremia, all infected cats had significant changes in mucosal T cell populations, suggesting that a low dose challenge may be sufficient to test vaccine efficacy if mucosal pathology is used as a primary correlate of protection.

For obvious reasons there is great interest in individuals with transient or controlled HIV-1 infection (thoroughly reviewed by Shacklett)[47]. Some studies have suggested that seronegative persons with high risk of exposure to HIV-1 may avoid infection as a result of low viral dose exposure [30,31]. In the present study, peak viremia was of similar magnitude regardless of dose, and there were cats in each dose group that did not become viremic. Thus, while virus dose undoubtedly plays a role in the likelihood of infection and viremia, the relationship is not linear and individual immune responses may be critical.

Of course, determination of virologic status depends on the sensitivity of methods used and tissue compartments that are evaluated. Clinically measurable seroconversion was only evident in 1/8 non-viremic cats, suggesting that these cats have occult infection. In this study, we used whole bone marrow, which has been shown to be a site of latency in FIV and other retroviral infections [48-51], to isolate virus. This method has been shown to be more sensitive in detecting low levels of retrovirus than standard real-time PCR techniques used to identify proviral integration in PBMC [52]. If standard clinical techniques had been used to determine the presence of infection, 6/17 cats would have been categorized as exposed and seronegative. As postulated by several authors and supported by our results, occult or latent infection, controlled by innate and cell-mediated immunity may occur more frequently in highly-exposed individuals than is currently recognized [30,31,53-56].

This study also comprehensively evaluated innate, cell-mediated and humoral immunity. NK cells are an important innate immune defense, particularly against intracellular pathogens [57], as they recognize virus infected cells without requiring costimulatory signals from other immune system cells, such as dendritic cells. Given their importance in clearing viral infection, we assessed the prevalence of total NK, classic (CD56+CD3-) and NKT cells in PBMC, lymph node and tissues. Decreased NK cell percentages, in general, and NKT cells, specifically, were associated with FIV infection while classic NK cells were preserved in non-viremic cats. A significant negative correlation was identified for CD56+CD3- NK cells and viremia that suggests innate immunity may play a greater role in control of acute retroviral infection than previously believed. Our results are consistent with prior observations of peripheral CD56+ NK cell loss in HIV-infected patients [58]. These results are supported by a study that showed increased NK cell function in highly-exposed seronegative injecting Vietnamese drug users as compared to individuals who eventually seroconverted [59]. However, a recent study that examined *in vitro *NK cell function of elite controllers suggested a more limited role for NK cells in control of viral replication [60]. A limitation of the present study is that the effector function of NK and CD8+ T cells was not evaluated using an in vitro killing assay. It is also important to note that increased presence of a specific population, e.g. NK cells, does not necessarily correlate with their ability to kill virally infected cells. Therefore, additional evaluation of NK cell frequency and function in HEPS, EC and LTNP is needed to better understand their role and the mechanisms used to control viral replication.

As numerous studies have shown an important role for cell-mediated immunity in HIV-1 control, we anticipated T cell function would be correlated with reduced viremia. However, correlation of IFN-γ and IL-2 production by CD4+ and CD8+ T cells yielded surprising results. A positive correlation to viremia was found for LPL CD4+ T cell production of IL-2 in response to Gag stimulation, with a similar trend observed for CD8+ LPL producing IL-2. In addition, while trends for inverse correlation with viremia were present for draining lymph node and LPL responses to Env stimulation, none were significant. This is in spite of significant differences in cytokine production when comparing viremic and non-viremic cats. Further, tissue specific cytokine responses in LPL were evident. Viremic cats were more likely to produce IFNγ and IL-2 against Gag peptides, whereas non-viremic cats produced IFNγ and IL-2 against Env peptides. However, these responses were insufficient to prevent the loss of CD4+ LPL and CD8+ IEL in non-viremic cats. However, we cannot dismiss the possibility that these cytokine responses may have helped reduce mucosal viral reservoirs, preventing widespread viral dissemination and viremia. As has been previously reported, control of viremia or lower viral set points are typically associated with the production of IFNγ and IL-2 within the same cell, or are typically associated with the continued ability to produce IL-2 upon stimulation [7,61,62]. Our results, similar to Pahar et al. [63] suggest that control of viremia cannot be directly correlated with cell-mediated immunity. Further, Barry et al. [64] showed that depletion of CD8+ T cells in non-pathogenic infection of sooty mangabey monkeys did not play a role in control of viral replication. In addition, others have shown in HIV+ patients that cell-mediated immunity did not correctly predict the outcome of infection and AIDS-free survival time [65,66]. Collectively, these results suggest that robust cell-mediated immune responses may not be an accurate correlate of protection.

Another surprising result was the strong positive correlation between anti-Env antibody production and the level of viremia. Studies of HIV-1 infected patients and SIV-infected non-human primates have divergent results with respect to anti-Env antibody responses and their role in altering progression of disease course. Some have shown that individuals with profound antibody responses progressed rapidly to AIDS [67-69]. However, other reports have suggested either a protective association of anti-Env antibodies with HIV-1 disease progression or no discernable pattern at all [70-74]. Our results showed that anti-Env and to a lesser degree anti-Gag antibody responses were associated with measurable infection, rather than occult infection. One possible explanation for this result is that significant levels of antibody were produced as a result of poor control by innate and cell-mediated immunity. Another possibility is that although antibodies were produced at high titers, they may not have been capable of virus neutralization.

Several studies of mucosal pathogenesis in HIV-1 infected humans and SIV infected macaques have focused on depletion of CD4+ LPL, as they are a primary target of infection [75-78]. However, it has been suggested that mucosal immune dysfunction may not be due solely to CD4+ LPL depletion. It has been shown that microbial translocation is a source of chronic antigenic stimulation in HIV infection [79], and that epithelial barrier dysfunction is evident in cellular and molecular processes prior to seroconversion [80]. Given the paucity of studies evaluating the important effector T cell population present in the epithelium, IEL were evaluated in conjunction with LPL in this study. Our results demonstrated that losses of IEL were as significant as the loss of CD4+ LPL. We have previously shown that CD8+ IEL are significantly depleted as early as one day following FIV infection [81]. The results of the present study suggest these early losses are not transient and occur specifically in the CD8αβ+ IEL. The combination of their loss with the loss of CD4+ LPL suggests that multiple immunologic factors may be involved in AIDS-associated enteropathy.

A key observation in this study was that all cats, regardless of initial viral dose, experienced profound acute losses in mucosal lymphocyte populations. In a previous study we demonstrated that protected vaccinated cats had mucosal immune populations similar to control cats one year after FIV infection, while unvaccinated cats that were non-viremic had disruptions in their mucosal lymphoid compartment [52]. In addition, non-pathogenic SIV infection of non-human primates has also shown that an initial loss of mucosal lymphocytes occurs, but when examined at later time points, these populations have been restored [82]. Collectively, these findings suggest that mucosal lymphocytes are the most sensitive indicator of infection as they are disrupted regardless of initial viral dose and seroconversion status, indicating that vaccine studies could indeed use preservation of IEL and LPL populations as a correlate of protection.

## Conclusions

In summary, this study provides valuable insight into the immune responses associated with early viral control in FIV infection. We found that NK cells may play a greater role in acute viral control than previously believed; however, other immune responses were associated with the ability of some of these cats to control viremia, and prevent seroconversion. We also show that more attention must be directed to dissecting immune responses not previously addressed in acute pathogenesis, with particular attention to innate immunity. Further, we identified the intestinal mucosa as a very sensitive indicator of retroviral infection that is independent of viral dose and seroconversion. Collectively, our data suggest that low dose challenge may be sufficient to test vaccine efficacy when considering mucosal immune integrity as a primary correlate of protection.

## Methods

### Animals and challenge inoculums

Twenty-three specific pathogen free (SPF) cats were obtained from Liberty Labs (Liberty, NY), group housed and cared for in accordance with AAALAC standards and IACUC guidelines. FIV-infected female cats included high dose (n = 6), middle dose (n = 6) and low dose (n = 5); control cats (n = 6) included 4 female and 2 neutered males. Age was six to eighteen months at euthanasia. Cats were infected with cell-associated and cell-free NCSU_1_, a FIV pathogenic sub-group A virus [35]. Cell-associated inoculum was created by intravenously inoculating a SPF cat with FIV-positive cells and harvesting all lymph nodes, spleen, and thymus after six weeks. Lymphocytes obtained were cultured for 7 days to increase the proportion of FIV-positive cells, and then cryopreserved. Supernatants from cultured cells were used to purify cell-free stocks. Cats were intravaginally inoculated with 3.75 × 10^5 ^FIV positive cells and 9.75 × 10^4 ^TCID_50 _cell-free FIV (high dose), 1.88 × 10^5^, FIV positive cells and 4.87 × 10^4 ^TCID_50 _cell-free FIV (middle dose) or 9.3 × 10^4 ^FIV positive cells and 2.43 × 10^4 ^TCID_50 _cell-free inoculum in RPMI, or were unexposed controls. Briefly, cats were sedated and placed in sternal recumbency with a small rolled towel placed under their caudal abdomen to elevate the reproductive tract. Cell-associated and cell-free viral inoculums were combined immediately prior to administration in a sterile microcentrifuge tube. The inoculum was atraumatically deposited on the mucosal surface of the vaginal vault using using a pipettor with a blunt polypropylene pipette tip and was completely absorbed in approximately five minutes.

Inoculum dose was determined based upon the results of several mucosal inoculation studies. We have previously used a combined dose of 7.5 × 10^4 ^FIV positive cells and 7.5 × 10^4 ^TCID_50 _cell-free and infected 6/10 control cats [74]. Other studies using the NCSU_1 _showed that all cats vaginally inoculated with 10^4 ^- 10^6 ^FIV-positive cells became FIV-positive, whereas those infected with 10^3 ^- 10^2 ^FIV-positive cells were either negative or latently positive [56]. Another study employing mucosal infection using a clade B virus strain, found that cats inoculated with 2.0 × 10^5 ^but not 2.0 × 10^3 ^FIV-positive cells were infected three weeks post-inoculation [43]. Given the results of these studies, and a prior pilot study (data not shown), inoculum doses were chosen for this study, with approximately one log difference in dose from the high to low dose inoculums.

### Sample collection and processing

Plasma, serum and vaginal wash fluids were collected at weeks 0, 2, 4, 6, and 8 post-infection and processed [83]. Peripheral blood mononuclear cells (PBMC), prescapular lymph node (PLN), mesenteric lymph node (MLN), medial iliac lymph node (ILN), spleen, bone marrow and distal jejunum were harvested at necropsy. Blood for PBMC isolation was collected at weeks 0, 2, 4, 6, and 8 in ACD tubes, and isolated using Histopaque (Sigma, St Louis, MO) density centrifugation [52]. Lymph nodes, thymus [84] and spleens [85] were processed as previously described. Bone marrow collected from the femur was dissociated using mesh screens. After washing, pellets were lysed with ammonium chloride lysis buffer, washed twice and counted. Intraepithelial lymphocytes (IEL) and lamina propria lymphocytes (LPL) were isolated from distal jejunum following excision of Peyer's patches and lymphoid follicles, as previously described [84].

### Real-time FIV RNA PCR

Real-time PCR to detect viral RNA in plasma was performed as previously described [86], with minor modifications. Real-time PCR was run on a Biorad MyIQ using continuous RT-PCR at 48°C for 30 minutes, 95°C for 10 minutes, followed by 45 cycles of 95°C for 10 seconds and 57.5°C for 1 minute. The limit of detection for this assay is ≤ 10 copies per 45 μl of plasma.

### Real-time FIV DNA PCR

Real time PCR was performed to quantify FIV provirus [87] using previously described primers and probe [86]. DNA was extracted using the DNeasy Tissue Kit (Qiagen, Valencia, CA). Real-time PCR reaction contained 300 nM forward primer, 400 nM reverse primer, and 80 nM probe, ABI Universal Mastermix (Applied Biosystems, Foster City, CA), water and DNA sample. The feline genome contains one copy of the CCR5 sequence and was used to normalize FIV copy number. The CCR5 primers (forward 5'-ACGTCTACCTGCTCAACCTGG-3', reverse 5'-ACCGTCTTACACATCCCATCCC-3') and probe (FAM-5'-TCCGACCTGCTCTTCCTCTTCACCCTCC-3') were designed using Beacon Designer. PCR reactions for CCR5 were run on a separate plate using 200 nM forward primer, 500 nM reverse primer, and 200 nM probe, ABI Universal Master mix, water and DNA sample. FIV and CCR5 plates were run sequentially on the same instrument. All standard dilutions, controls and samples were run in duplicate. The limit of detection was ≤10 copies of FIV per 1 μg DNA.

### Virus isolation

Cryopreserved bone marrow samples were thawed, washed, counted and 1.5 × 10^5 ^co-cultured with 3.0 × 10^5 ^FCD4-Ecells or Mya-1 cells, both of which are FIV susceptible cell lines, in triplicate in LBT medium supplemented with 100U/ml recombinant human IL-2 (AIDS Research and Reference Reagent Program, Division of AIDS, NIAID, NIH: contributed by Hoffman-La Roche Inc.). Supernatants were analyzed for FIV p24 by antigen capture ELISA at 16 and 20 days of culture [52]. In addition, cells were analyzed for intracellular viral antigen by FACS at 20 days of culture using the BD Cytofix/Cytoperm kit and FITC conjugated anti-FIV monoclonal antibody 43-1B9.

### Monoclonal antibodies

Monoclonal antibodies (mAb) used for cell surface staining were directly conjugated to FITC, PE, biotin, PerCP, APC, PECy7, or Alexa 700. Previously described antibodies included anti-CD4 (3-4F4), anti-CD8α (3.357), Streptavidin-PerCP, and Streptavidin-APC [84]. Other antibodies included anti-feline CD8β and Streptavidin-Pe-Cy7 (Southern Biotechnology, Birmingham, AL); anti-CD56 (clone C5.9, Dako, Carpinteria, CA); anti-tumor necrosis factor alpha (TNFα, clone 6401.1111, BD Biosciences, San Jose, CA); anti-interleukin-2 (IL2, clone MQ1-17H12, Biolegend, San Diego, CA), and anti-interferon gamma (IFNγ, clone E4A3B9) made in our laboratory. Anti-feline CD3 (clone NZM1), a generous gift from T. Miyazawa (Univ. of Tokyo, Japan) detected with anti-mouse IgG3 APC (#115-135-209, Jackson Immunoresearch, West Grove, PA). Staining and flow cytometric analysis were performed as previously described [84]. Samples were fixed with 4% paraformaldehyde, collected on a BD LSRII (BD Immunocytometry Systems, San Jose, CA) flow cytometer, and analyzed with BD FACS Diva software.

### Intracellular cytokine production

PBMC, spleen, PLN, ILN, IEL and LPL were cultured overnight in LBT medium without stimulation. Cells were then stimulated with either Gag or Env peptide pools, Con A, or media alone for six hours, with 1.5 μl/ml of 1x Monensin (Biolegend, San Diego, CA) present for the final five hours. Following culture, cells were washed with PBS and stained with antibodies to CD4 and CD8, washed, and fixed with 4% paraformaldehyde (PFA) for ten minutes, then washed and resuspended in PBS/2%FBS, covered and stored at -4°C. Within 24-48 hours of PFA fixation, cells were washed with Perm/Wash Buffer (BD Cytofix/Cytoperm), stained with antibodies to IFNγ, IL-2, and TNFα for 30 minutes, washed and analyzed immediately, collecting 100,000 gated events whenever possible. For analysis, cell populations were initially gated to identify the viable leukocyte population on FSC and SSC. Using this gate, CD4+ and CD8+ T cells were individually gated and CD4+ and CD8+ T cells producing IL-2, IFNγ and TNFα in response to Gag or Env peptide stimulation identified (Additional file 1). The percentage of T cells responding to stimulation was calculated by subtracting the cytokine response detected in cells from the same sample stimulated with media alone.

### FIV p24 antibody chemiluminescent ELISA

Chromalux™ HB Luminescent Assay microplates (Dynex Technologies Inc., Chantilly, VA) were coated with 1.0 μg/ml p24-GST fusion protein [88] and ELISA completed as previously described [89] with minor modifications. Blocking buffer contained CBC buffer with 5% nonfat dry milk and 0.1% Kathon. ELISA wash buffer (EWB) contained PBS, 0.05% Tween-20, and 0.1% Kathon. Samples were serially diluted in sample diluent (PBS, 5% non-fat dry milk, 5% goat serum, 0.05% Tween-20 and 0.1% Kathon), added to ELISA plates at 100 μl/well, covered and incubated for three hours at 37°C, then washed six times with EWB. Antibody was detected with goat anti-cat IgG (Bethyl Labs, Montgomery, TX) or goat anti-IgA (Serotec, Raleigh, NC) diluted 1:80,000 and 1:3,000 respectively in sample diluent, covered, incubated one hour at 25°C, then washed six times with EWB. Plates were developed with 100 μl/well Pierce SuperSignal ELISA Femto Chemiluminescent substrate #37074 and read five minutes after addition of substrate (Perkin-Elmer Victor 3 1420 Multilabel Counter). Antibody titers were considered positive if they were two-fold greater than the naïve serum/vaginal wash sample for the same individual.

### FIV Env antibody chemiluminescent ELISA

Anti-envelope responses were measured using a previously described envelope fusion protein (gp95-FC) [90]. Anti-Env ELISA was completed as described for anti-p24 antibody ELISA with minor modifications. Blocking buffer contained CBC buffer with 3% non-fat dry milk, 5% goat serum, and 0.1% Kathon. Sample diluent contained PBS, 1% BSA, 1% non-fat dry milk, 2.5% human serum, 0.05% Tween-20 and 0.1% Kathon. Plates were coated with 100 μl/well of gp95-FC at 2.5 μg/ml in sample diluent. Sample dilutions were incubated for 30 minutes at 25°C prior to loading 100 μl/well to ELISA plates. Secondary antibodies for anti-IgG and anti-IgA detection were diluted to 1:60,000 and 1:3,000 respectively.

### Statistical analysis

Comparison of viremic versus non-viremic FIV inoculated cats was completed using an unpaired t-test. Statistical analysis of differentially FIV-inoculated groups and control cats utilized a one-way analysis of variance (ANOVA) with a Tukey-Kramer multiple comparison post-test or nonparametric ANOVA (Kruskal-Wallis test) with Dunn's multiple comparison post-test. The choice of test was dictated by testing the assumptions necessary for parametric methods, such that if a parametric method was not appropriate, non-parametric testing was used. Significance was defined as p ≤ 0.05. Analyses were completed using GraphPad InStat version 3.05 (GraphPad Software, San Diego, CA).

## Competing interests

The authors declare that they have no competing interests.

## Authors' contributions

KH designed and coordinated the study, collected and processed biological samples, performed FACS assays and virus isolation, analyzed data, performed statistical analysis and drafted the manuscript. SR helped design the study, collected and processed biological samples, performed FACS assays and assisted in data analysis. EE collected and processed biological samples, performed PCR and ELISA assays and assisted in data analysis. GD helped design the study, critically reviewed the results and helped to draft the manuscript. All authors read and approved the final manuscript.

## Supplementary Material

Additional File 1 **Supplemental Figure 1. Gating strategy for intracellular cytokine assessment**. Samples stained for surface and intracellular antigens were gated based on forward and side scatter (A). The gated population was used to identify CD4+ and CD8+ T cells (B). Using either CD4+ or CD8+ T cells as the parent gate, specific staining for IFNγ (y-axis) and IL-2 (x-axis) are shown for unstimulated (C) and stimulated samples (D) from the same medial iliac lymph node. For analysis, the percent of cells staining positively in unstimulated samples was subtracted from stimulated samples to determine net cytokine production reported in Figure 6. Click here for file
