# Supplementary figures and images for: Acute mucosal pathogenesis of feline immunodeficiency virus is independent of viral dose in vaginally infected cats
Source: Retrovirology. 2010 Jan 19;7:2. doi: 10.1186/1742-4690-7-2 (PMC2835650; doi:10.1186/1742-4690-7-2)

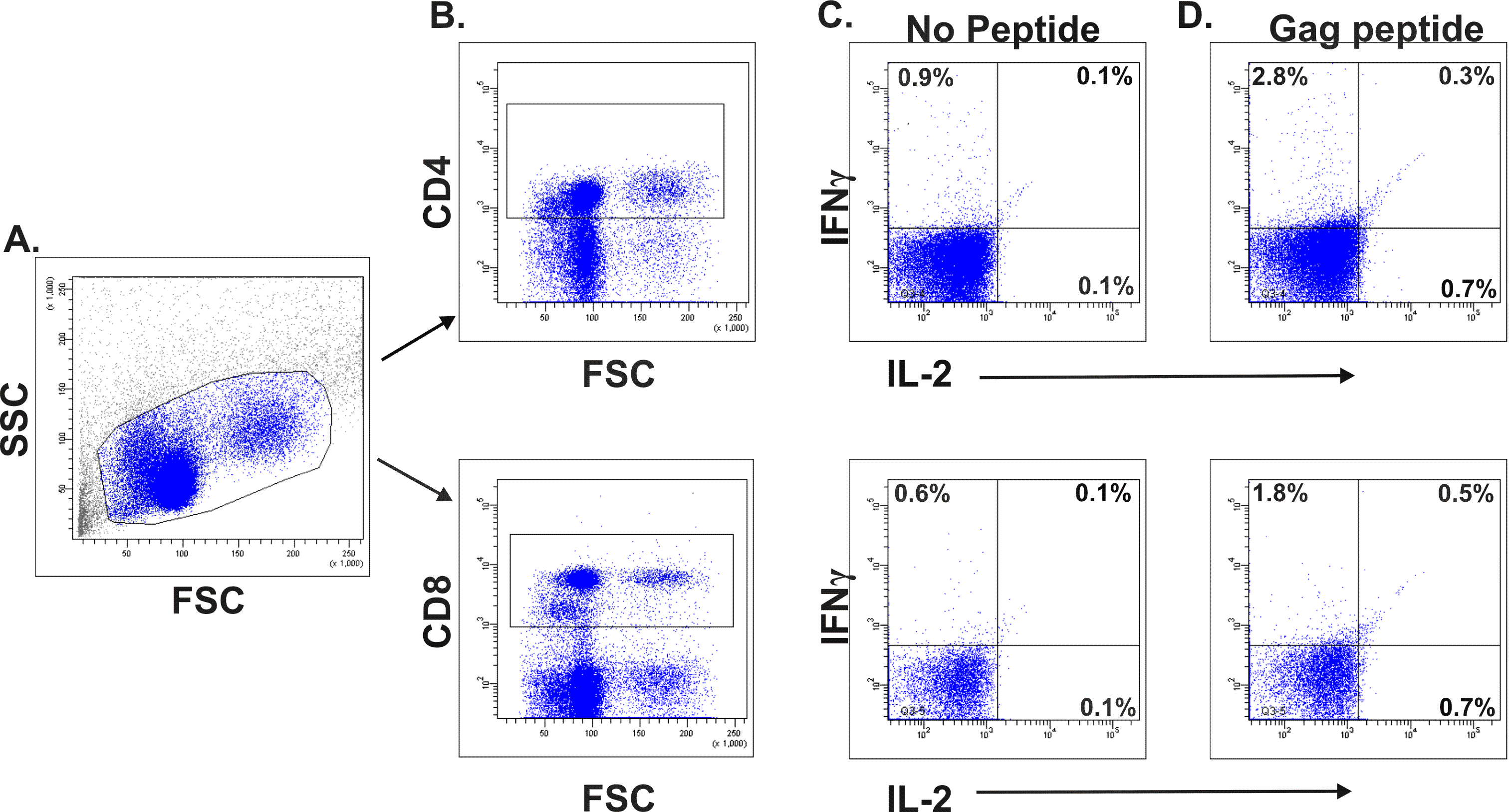

Supplement: Additional File 1 — Supplemental Figure 1. Gating strategy for intracellular cytokine assessment. Samples stained for surface and intracellular antigens were gated based on forward and side scatter (A). The gated population was used to identify CD4+ and CD8+ T cells (B). Using either CD4+ or CD8+ T cells as the parent gate, specific staining for IFNγ (y-axis) and IL-2 (x-axis) are shown for unstimulated (C) and stimulated samples (D) from the same medial iliac lymph node. For analysis, the percent of cells staining positively in unstimulated samples was subtracted from stimulated samples to determine net cytokine production reported in Figure 6. [file 1742-4690-7-2-S1.BMP]
